# Risk factors of early periprosthetic femoral fracture after total knee arthroplasty

**DOI:** 10.1186/s12891-021-04875-5

**Published:** 2021-12-02

**Authors:** Chaturong Pornrattanamaneewong, Akraporn Sitthitheerarut, Pakpoom Ruangsomboon, Keerati Chareancholvanich, Rapeepat Narkbunnam

**Affiliations:** 1 Department of Orthopaedic Surgery, Faculty of Medicine Siriraj Hospital, Mahidol University, 2 Wang Lang Road, Bangkok Noi, Bangkok, 10700 Thailand; 2 Department of Orthopaedic Surgery, Prajuabkirikhan Hospital, Bangkok, Thailand

**Keywords:** Risk factor, Periprosthetic fracture, Total knee arthroplasty, Age

## Abstract

**Background:**

Periprosthetic femoral fracture (PFF) is a serious complication after total knee arthroplasty (TKA). However, the risk factors of PFF in the early postoperative setting are not well documented. This study determines the risk factors of early PFF after primary TKA.

**Methods:**

This study recruited 24 patients who had early PFF within postoperative 3 months and 96 control patients. Demographic data (age, gender, weight, height, body mass index, Deyo-Charlson comorbidity index, diagnosis, operated side, underlying diseases and history of steroid usage intraoperative outcomes), intraoperative outcomes (operative time, surgical approach, type and brand of the prosthesis), and radiographic outcomes (distal femoral width; DFW, prosthesis-distal femoral width ratio; PDFW ratio, anatomical lateral distal femoral angle; LDFA, the change of LDFA, femoral component flexion angle; FCFA and anterior femoral notching; AFN) were recorded and compared between groups. Details of PFF, including fracture pattern, preoperative deformity, and time to PFF were also documented.

**Results:**

In univariate analysis, the PFF group had significantly older, right side injury, rheumatoid, dyslipidemia, Parkinson patients than the control group (*p* < 0.05). No cruciate-retaining design was used in PFF group (*p* = 0.004). Differences between the prosthetic brand used were found in this study (*p* = 0.049). For radiographic outcomes, PFF group had significantly lower DFW but higher PDFW ratio and postoperative LDFA than the control group (*p* < 0.05). While the change of LDFA, FCFA and AFN were similar between groups. The fracture patterns were medial condylar (45.8%), lateral condylar (25.0%) and supracondylar fracture (29.2%). The mean overall time to PFF was 37.2 ± 20.6 days (range 8–87 days). Preoperative deformity was significantly different among the three patterns (p < 0.05). When performed multivariate analysis using the logistic regression model, age was only an independent risk factor for early PFF. The cut-off point of age was > = 75 years, with a sensitivity of 75.0% and specificity of 78.1%.

**Conclusion:**

This study determined that age was the independent risk factors for early PFF. However, further well-controlled studies with a larger sample size were needed to address this issue.

## Introduction

Periprosthetic femoral fracture (PFF) is one of the serious complications after total knee arthroplasty (TKA). It is associated with significant morbidity, which requiring further procedures and additional cost [1]. The incidence of PFF has been reported ranging from 0.3–2.5% and typically located in the supracondylar region [2, 3]. Most of PFF occurs following a low energy trauma such as a simple fall [4]. However, it can also occur in patients without a history of trauma [3].

Several risk factors have been recognized, which can be arranged into the patient, the surgical, and implant-related factors. Patient factors include advanced age, female gender, rheumatoid arthritis (RA), neurologic diseases, osteoporosis, and chronic steroid use [3, 5, 6]. While some surgical or technical errors such as anterior femoral notching (AFN) may increase the risk of PFF [1]. In terms of implant-related factors, although Alden et al. [7] demonstrated an increased risk of intraoperative femoral fracture with posterior-stabilized (PS) design, this factor is still disputable for postoperative femoral fracture.

Most of the mentioned studies define the risk factors of PFF in the overall postoperative period. The risk factors of PFF in the early postoperative setting are not well reported. Thus, the purpose of this study is to find out the risk factors of early PFF after primary TKA. Our results may help the surgeon to create strategic planning for preventing this devastating complication.

## Methods

The institutional review board approved this study protocol and registered as TCTR20200226001. Between March 2008 and May 2019, the patients who underwent primary TKA in our institute and had PFF within 3 months after surgery were recruited (PFF group). We excluded the patients who had PFF related to high energy trauma. During the operation, we aimed to restore the neutral mechanical alignment in all patients. In order to reduce the differences of surgical techniques and instruments used in each time period, the patients who had no PFF within 3 months were selected as the followings; when we met the PFF case, two consecutive patients who underwent before and two consecutive patients who underwent after PFF case were recruited and collected as the control group. The exclusion criteria of both groups were the patients required stem or metal augmentation, intraoperative fracture or consequent intraoperative fracture from immediate postoperative radiograph, collateral ligament injury, periprosthetic joint infection, and incomplete data. All procedures were performed in accordance with relevant guidelines.

**Fig. 1:**
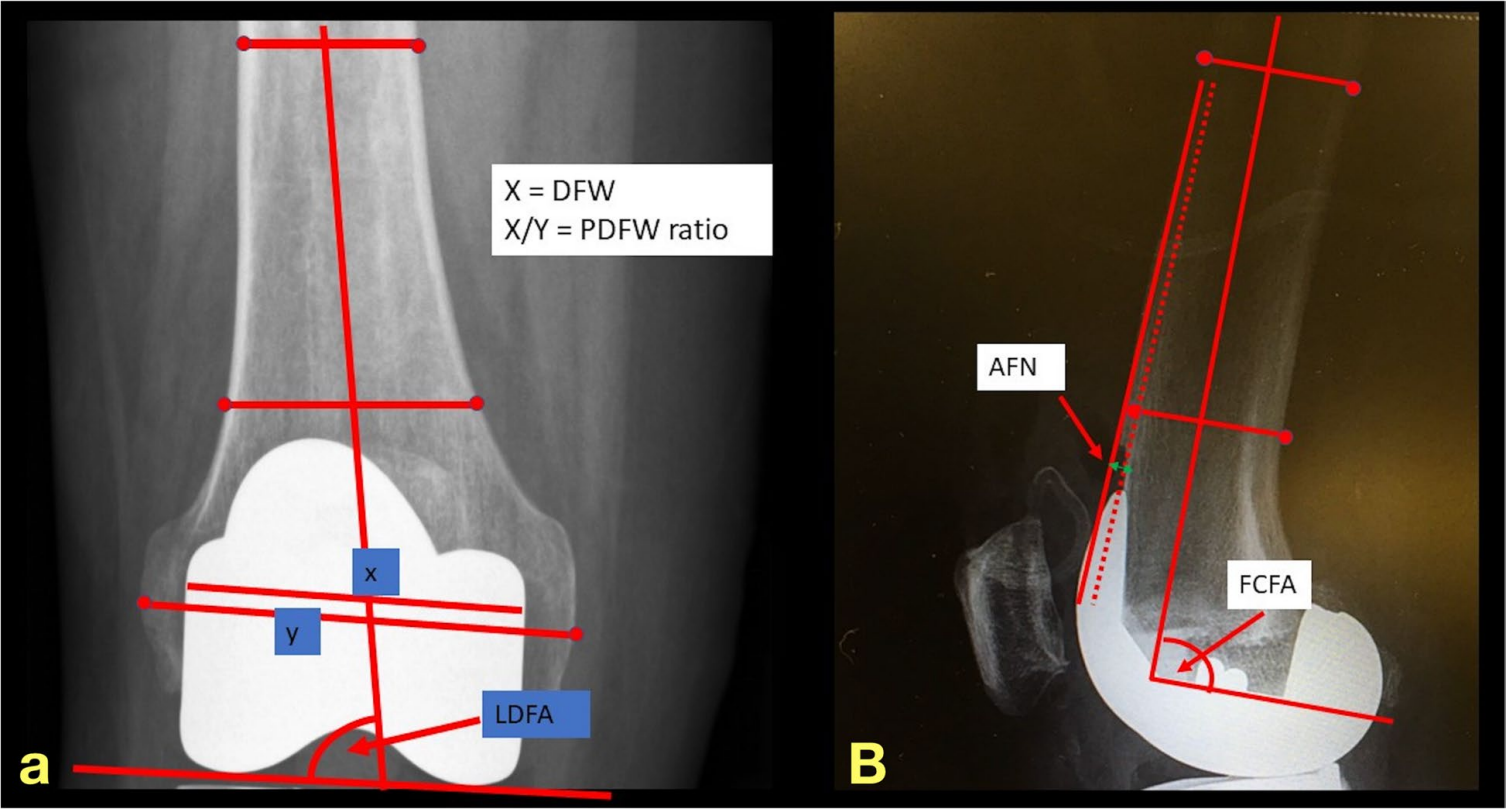
The measurements of knee radiographic outcomes from **a**) anteroposterior view (anatomical lateral distal femoral angle, LDFA; distal femoral width, DFW; and prosthesis-distal femoral width ratio, PDFW) and **b**) lateral view (femoral component flexion angle, FCFA; and anterior femoral notching, AFN)

Patients’ characteristics including age, gender, weight, height, body mass index (BMI), Deyo-Charlson comorbidity index (DCCI), diagnosis, operated side, underlying diseases, and history of steroid usage were collected. Operative time, surgical approach, type, and brand of the prosthesis were recorded as the intraoperative outcomes. For radiographic outcomes, preoperative anatomical lateral distal femoral angle (LDFA), and postoperative parameters including postoperative LDFA, distal femoral width (DFW), and prosthesis-distal femoral width (PDFW) ratio were measured from pre- and postoperative anteroposterior knee radiographs, respectively. To determine the deformity correction angle of distal femur, the change of LDFA was calculated from postoperative LDFA minus preoperative LDFA. The measurement method was described as the followings; First, the distal femoral joint line (DJL) was drawn using the line connecting the distal-most aspects of medial and lateral condyles of the distal femur or femoral component. The anatomical femoral axis (AFA) was defined as the line connecting two midpoints of the femoral shaft at 5 and 10 cm above the DJL. The LDFA was the lateral angle between the AFA and DJL. The change of LDFA was calculated from postoperative LDFA minus preoperative LDFA. The DFW was defined as the longest distance of the distal femur that parallels to the DJL. Furthermore, the PDFW ratio was the ratio of the mediolateral width of the femoral component to DFW. The femoral component flexion angle (FCFA) and anterior femoral notching (AFN) were measured on the lateral view of the knee radiograph. The sagittal femoral axis (SFA) was drawn using the line connecting two midpoints of the femoral shaft at 5 and 10 cm above the most distal part of the femoral component. The FCFA was the posterior angle between SFA and the sagittal plane of the femoral component. The AFN was measured as the depth between anterior femoral cortex and the anterior cut line of the distal femur [8] (Fig. 1). Details of PFF, including fracture pattern, preoperative deformity, preoperative anatomical femorotibial angle (aFTA), and time to PFF were also recorded.

### Statistical analysis

The statistical analysis was performed using SPSS program version 18.0 (SPSS Inc., Chicago, Illinois). In the univariate analysis, the Student t-test was used to compared continuous data between groups. Consequently, analysis of variance was used to compare continuous data among the different fracture patterns. The Chi-square or Fisher-exact test was used to compared categorical data in our study. To find out the independent risk factors for PFF, the univariate and multivariate analysis were then performed using the logistic regression model. All variables with *p*-value < 0.2 were entered into the model. The crude and adjusted odds ratio (OR) with 95% confidence interval (CI) were calculated. The statistical significance was considered if a p-value < 0.05.

## Results

**Fig. 2:**
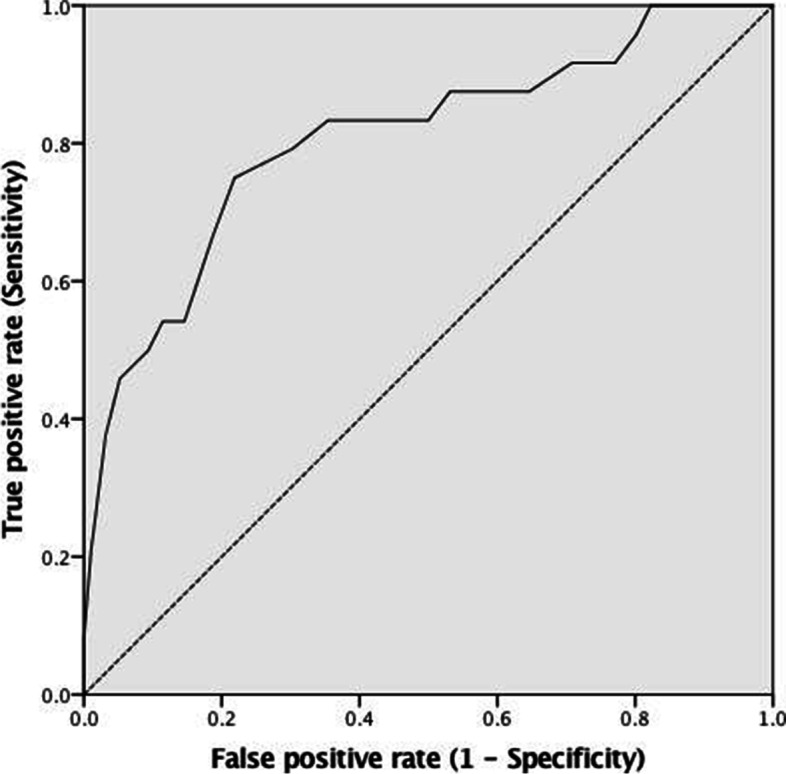
The receiver operating characteristics curve for prediction of early periprosthetic femoral fracture based on the age at index of total knee arthroplasty

A total of 24 PFF patients were included for analysis. Thus 96 controlled patients were selected in this study. The patients’ characteristics of both groups were shown in Table 1. The mean age in the PFF group was significantly higher than the control group (*p* < 0.001). The majority of overall patients were female. PFF group had more right-sided injury than the control group (*p* = 0.012). More RA patients were found in the PFF group (*p* = 0.039). The PFF patients had a significantly higher prevalence of underlying dyslipidemia and Parkinson’s disease than the control group (*p* = 0.040 and 0.007, respectively). The diagnosis of dyslipidemia was made when either type of lipid abnormalities was present (serum LDL-cholesterol > = 140 mg/dL, HDL-cholesterol < 40 mg/dL or triglycerides > = 150 mg/dL). The use of cruciate-retaining (CR) design was significantly lower in the PFF group (*p* = 0.004). A significant difference in the prosthesis brand used was also found between groups (*p* = 0.049). In radiographic outcomes, as shown in Table 2, the PFF group had significantly lower DFW than the control group (*p* = 0.001), while the PDFW ratio and LDFA were significantly higher in the PFF group (p = 0.004 and 0.026, respectively). After using logistic regression model, age, side, dyslipidemia, DFW, PDFW ratio and LDFA were identified as the significant factors in univariate analysis. However, when entering the variables with *p* < 0.2 into the multivariate analysis, age was only significantly independent risk factor for PFF (Adjusted OR 0.85, 95%CI 0.76–0.94, *p* = 0.002) (Table 3). To identify the cut-off point for age, we performed the post-hoc analysis using the receiver operating characteristics (ROC) curve (Fig. 2). The area under the curve was 0.806 (95% CI, 0.700 to 0.912, *p* < 0.001). Finally, the cut-off point of age > =75 years that provided the highest summation of sensitivity and specificity was determined. There were 18 PFF and 21 control patients In the patients aged > = 75 years. The sensitivity, specificity, positive and negative predictive value were 75.0, 78.1, 46.2 and 92.6%, respectively.

**Table 1:** Patients’ characteristics

Characteristics	PFF group (*n* = 24)	Control group (*n* = 96)	*p*-value
Age (yr)	77.1 ± 6.7	69.0 ± 7.2	< 0.001*
Gender (female,%)	22 (91.7%)	84 (87.5%)	0.733
Weight (kg)	63.5 ± 11.2	66.5 ± 12.8	0.287
Height (cm)	152.6 ± 7.6	154.9 ± 7.8	0.205
BMI (kg/m^2^)	27.3 ± 4.6	27.7 ± 4.8	0.701
Side (right,%)	20 (83.3%)	53 (55.2%)	0.012*
DCCI (scores)	0.5 ± 0.9	0.3 ± 0.5	0.348
Diagnosis (%)			
Osteoarthritis	22 (91.7%)	96 (100.0%)	0.039*
Rheumatoid arthritis	2 (8.3%)	0 (0.0%)	
Underlying diseases (%)			
Diabetes	5 (20.8%)	25 (26.0%)	0.598
Hypertension	18 (75.0%)	73 (76.0%)	0.915
Dyslipidemia	14 (58.3%)	34 (35.4%)	0.040*
Cardiovascular disease	2 (8.3%)	5 (5.2%)	0.626
Thyroid disease	1 (4.2%)	1 (1.0%)	0.361
Parkinson disease	3 (12.5%)	0 (0.0%)	0.007*
Steroid use (%)	1 (4.2%)	0 (0.0%)	0.200
Surgical approach (%)			
Medial parapatellar	23 (95.8%)	95 (99.0%)	0.361
Midvastus	1 (4.2%)	1 (1.0%)	
Operative time (min)	81.5 ± 23.4	79.9 ± 30.2	0.201
Prosthesis design (%)			
Cruciate-retaining	0 (0.0%)	24 (25.0%)	0.004*
Posterior-stabilized	24 (100.0%)	72 (75.0%)	
Prosthesis brand (%)			
Zimmer	20 (83.3%)	63 (65.6%)	0.049*
Depuy	3 (12.5%)	31 (32.3%)	
Stryker	1 (4.2%)	0 (0.0%)	
Smith Nephew	0 (0.0%)	2 (2.1%)	

*PFF* periprosthetic femoral fracture, *BMI* body mass index, *DCCI* Deyo-Charlson comorbidity index

*p-value < 0.05

**Table 2:** Radiographic outcomes

Outcomes	PFF group (*n* = 24)	Control group (*n* = 96)	*p*-value
LDFA (º)			
-Preoperative	81.8 ± 3.4	81.9 ± 2.2	0.855
-Postoperative	86.2 ± 2.0	85.2 ± 1.9	0.026*
-Change of LDFA	4.4 ± 4.3	3.3 ± 2.3	0.227
DFW (mm)	80.4 ± 5.3	85.7 ± 7.0	0.001*
PDFW ratio	0.79 ± 0.01	0.75 ± 0.05	0.004*
FCFA (º)	88.4 ± 6.0	87.8 ± 4.1	0.635
AFN (%)	2 (8.3%)	1 (1.0%)	0.102
AFN (mm)	0.3 ± 1.1	0.1 ± 0.5	0.301

*PFF* periprosthetic femoral fracture, *LDFA* anatomical lateral distal femoral angle, *DFW* distal femoral width, *PDFW* prosthesis-distal femoral width, *FCFA* femoral component flexion angle, *AFN* anterior femoral notching

*p-value < 0.05

**Table 3:** Logistic regression analysis

Variables	Univariate analysis			Multivariate analysis		
	Crude OR	95% CI	*p*-value	Adjusted OR	95% CI	*p*-value
Age	0.83	0.76–0.91	< 0.001*	0.85	0.76–0.94	0.002*
Side						
-Right	1	Reference		1	Reference	
-Left	4.06	1.29–12.77	0.017*	3.50	0.86–14.2	0.080
Diagnosis						
-Osteoarthritis	1	Reference		1	Reference	
-Rheumatoid arthritis	0	NA	0.999	NA	NA	NA
Dyslipidemia						
-No	1	Reference		1	Reference	
-Yes	0.39	0.16–0.98	0.044*	0.53	0.15–1.87	0.325
Parkinson disease						
-No	1	Reference		1	Reference	
-Yes	0	NA	0.999	NA	NA	NA
Prosthetic design						
-Cruciate-retaining	1	Reference		1	Reference	
-Posterior-stabilized	0	NA	0.999	NA	NA	NA
Prosthetic band						
-Zimmer	1	Reference		1	Reference	
-Depuy	3.28	0.91–11.89	0.071	4.74	0.87–25.84	0.072
-Stryker	0	NA	1.000	0	NA	1.000
-Smith Nephew	NA	NA	0.999	NA	NA	0.999
Postoperative lateral distal femoral angle	1.31	1.03–1.69	0.030*	1.17	0.84–1.61	0.353
Distal femoral width	1.17	1.06–1.28	0.001*	1.16	0.98–1.36	0.085
Prosthesis-distal femoral width ratio	0	0.00–0.02	0.006*	0.36	NA	0.917
Anterior femoral notching						
-No	1	Reference		1	Reference	
Yes	0.12	0.01–1.34	0.084	0.12	0.00–59.61	0.500

*OR* odds ratio, *CI* confidence interval, *NA* not applicable

*p-value < 0.05

Table 4 demonstrated the analysis of the pattern of PFF; the most common location was medial condylar fractures (11 cases, 45.8%). While 7 (29.2%) and 6 (25.0%) cases had supracondylar and lateral condylar fractures, respectively. Significant differences of preoperative deformity and aFTA were found among three patterns of PFF (*p* = 0.036 and 0.036, respectively). All lateral condylar fractures were associated with preoperative valgus deformity. Nevertheless, most supracondylar fractures were related to preoperative varus deformity. The mean overall time to PFF was 37.2 ± 20.6 days (range 8–87 days). Although supracondylar fractures had a longer time than the others, these differences did not reach statistical significance (*p* = 0.266).

**Table 4:** Patterns and data of periprosthetic fracture

Data	Medial condylar fracture	Lateral condylar fracture	Supracondylar fracture	*p*-value
Number (%)	11 (45.8%)	6 (25.0%)	7 (29.2%)	NA
Preoperative deformity (%)				
Varus	5 (45.5%)	6 (100.0%)	6 (85.7%)	0.036*
Valgus	6 (54.5%)	0 (0.0%)	1 (14.3%)	
aFTA (º)	176.3 ± 10.3	189.8 ± 5.3	183.3 ± 11.5	0.036*
Time to fracture (days)	33.3 ± 16.1	31.8 ± 20.9	48.0 ± 25.3	0.266

*aFTA* anatomical femorotibial angle, *NA* not applicable

*p-value < 0.05

## Discussion

Our study was the first investigation that ascertained the risk factors of PFF in the early postoperative period within 3 months. In univariate analysis, our significant factors, including age, RA, and neurologic disease, were similar to previous studies [3, 5, 6]. Although several pre-existing neurological diseases, including epilepsy, Parkinson’s disease, and poliomyelitis were proposed as the risk factors [9], our study only had Parkinson patients. These diseases were related to an increase in falls. Regarding the operated site, Zainul-Abidin et al. [1] reported left-sided surgery was a significant risk factor. However, the opposite side was reported in our study. The relevance of this factor was still unexplained.

In terms of prosthetic design, our study revealed no CR design used in PFF group. Besides, we also demonstrated that PS design was the risk factor for early PFF, which this factor had never been reported. Alden et al. [7] reviewed 49 intraoperative femoral fractures from 17,389 primary TKA. These fractures could occur during exposure, bone preparation, and trialing of the component. They found that the PS design had a higher risk of intraoperative femoral fracture than CR design. The relative risk was 4.74. From their conclusions, we hypothesized that the intercondylar box cut of PS design might cause stress riser or intraoperative occult fracture. It might lead to early PFF in some patients.

The relationship between prosthetic and distal femoral bone sizes was another concern that we investigate. In univariate analysis, smaller distal femoral bone and larger prosthesis compared to bone or PDFW ratio were the risk factors of PFF. However, these factors were not significant when fitting to the multivariate regression model. For femoral component positioning, the correlation between malalignment and PFF was not well documented. Although LDFA in PFF group was significantly higher than the control group, the amount of difference was not clinically important.

The most significant finding of our study existed that age was independent risk factors of early PFF. The cut-off point of age was > = 75 years. Compared to previous literatures, the systematic review of Canton et al. [10] revealed that advanced age was the main patient related risk factor for periprosthetic knee fracture, particularly because of its association with higher risk of fall and with osteoporosis. Considering the cut-off point of age, Singh et al. [11] collected the 17,633 primary TKA from Mayo Clinic Total Joint Registry data and found that only age was significantly associated with risk of periprosthetic knee fracture. Age > 80 years was associated with higher risk than ages 61–70 (Hazard ratio, HR 1.9, 95% CI 1.1–3.1, *p* = 0.02) and 71–80 years (HR 1.7, 95% CI 1.0–2.9, *p* = 0.04). Nevertheless these literatures studied on postoperatively periprosthetic knee fracture, not focused on the early PFF.

**Fig. 3:**
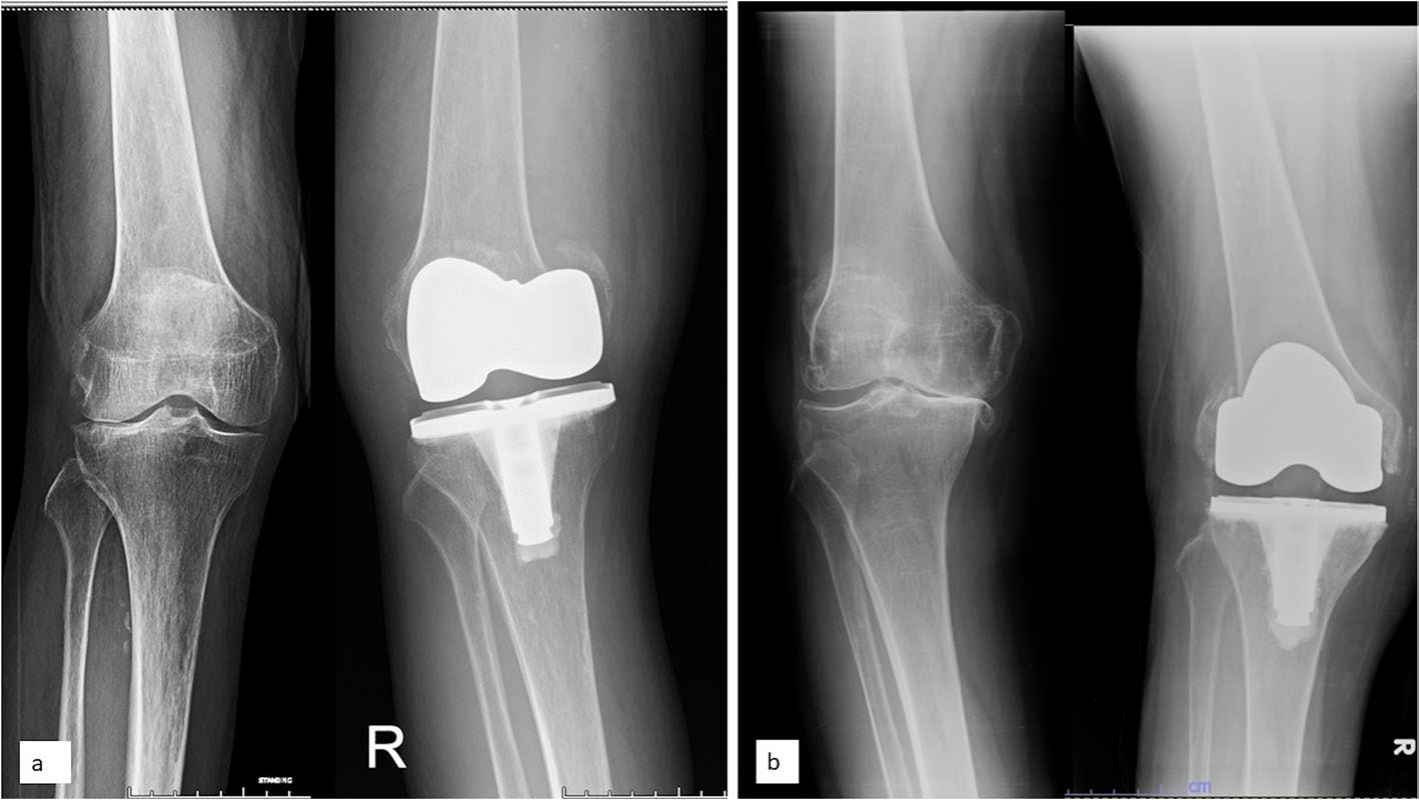
Preoperative and postoperative anteroposterior knee radiographs of two patients demonstrating **a**) medial condylar fracture and **b**) lateral condylar fracture, respectively

The most common mechanism for a supracondylar fracture was a low-velocity fall. Although AFN more than 3 mm with a sharp corner at the proximal end of a femoral component provided the highest stress concentration in a biomechanical study [12], a recent prospective clinical trial could not show the correlation between AFN and supracondylar fracture [13]. Our study also could not determine AFN as a risk factor. For the condylar fracture, Vestermark et al. [3] found that seven patients sustained a condylar fracture in the acute postoperative setting. Five patients had preoperative valgus deformity and sustained fracture of unloaded medial condyle. The other two patients had preoperative varus deformity and sustained fracture of unloaded lateral condyle. The authors called this type of fracture as “early femoral condyle insufficiency fracture”. Comparable to our study, all lateral condylar fractures had preoperative varus deformity. While 54.5% of medial condylar fractures had preoperative valgus deformity (Fig. 3), we believed that insufficiency fracture might explain this phenomenon. Nevertheless, the remaining 45.5% of medial condylar fracture was still associated with preoperative varus deformity. Due to a fracture of the loaded medial condyle, it should be caused by a technical error during surgery.

In clinical application, appropriate surgical exposure, avoiding excessive bow cut if PS design was used, gentle trial reduction, and prosthesis insertion were essential for minimizing this complication. Because early PFF was not found in the CR design used, we recommended that the use of this design might be beneficial for high-risk patients. For surgeons who preferred PS design, intraoperative surveillance for occult fracture, and preparation of the backup femoral stem should be performed. Likewise, we thought that the prophylactic femoral stem insertion was another strategy to prevent PFF. A finite element study revealed that periprosthetic stress was reduced through the use of a femoral stem. It might help mitigate PFF risk [14]. However, big high-quality data was necessitated for stratifying or scoring the risk factors and identifying the appropriate patients.

There were several limitations to our study. First, our research was a retrospective design; retrieving some of the information that we need might be troublesome. Osteoporosis was one of the most critical factors that contribute to PFF risk. Bernatz et al. [15] reported that one-quarter of total joint arthroplasty patients met the criteria to receive osteoporosis medications. This lack of preoperative osteoporosis screening and treatment has also happened in our study. Second, our study’s small sample size might decrease statistical power to detect the other significant risk factors. The collection of more data from multicenter might get more meaningful results. Additionally, in case of small sample size, the matched case-control designed study was recommended. However, we thought that some baseline patients ‘characteristics might be the significantly independent factors. We therefore decided to match case and control using the time period instead. Third, most patients in this study were female that had a higher risk. Thus, our results could not be applied to male patients. Fourth, all radiographic outcomes were measured from short radiographs because we had not sent the full-length radiographs routinely in the early postoperative period. However, Alzahrani et al. [16] illustrated the good to the excellent correlation of short and full-length radiographs. They also suggested that short radiographs could be an appropriate substitute for full-length radiographs for evaluating postoperative coronal alignments. Lastly, although we tried to detect the consequent intraoperative PFF from immediate postoperative radiographs, it was challenging to distinguish the early PFF from occult intraoperative PFF.

In conclusion, we found that age was independent risk factors for early PFF. The cut-off point of age was > = 75 years, with a sensitivity of 75.0% and specificity of 78.1%. The further well-controlled studies with large sample size were needed to elucidate this research question. It would support us in doing strategic planning for preventing this complication.

## Data Availability

Requests for data not shown in the body of this manuscript can be made to the corresponding author.
